# *N*-(2,5-Dimethyl­phen­yl)benzene­sulfonamide

**DOI:** 10.1107/S1600536809041841

**Published:** 2009-10-17

**Authors:** B. Thimme Gowda, Sabine Foro, P. G. Nirmala, Hartmut Fuess

**Affiliations:** a Department of Chemistry, Mangalore University, Mangalagangotri 574199, Mangalore, India; b Institute of Materials Science, Darmstadt University of Technology, Petersenstrasse 23, D-64287, Darmstadt, Germany

## Abstract

In the title compound, C_14_H_15_NO_2_S, the dihedral angle between the aromatic rings is 40.4 (1)° relative to each other. In the crystal, inversion dimers linked by pairs of N—H⋯O hydrogen bonds occur.

## Related literature

For the preparation of the title compound, see: Gowda *et al.* (2005 ▶). For related structures, see: Gowda *et al.* (2008**a* ▶,b* ▶; 2009 ▶). For bond-length data for other aryl sulfonamides, see: Gelbrich *et al.* (2007 ▶); Perlovich *et al.* (2006 ▶).
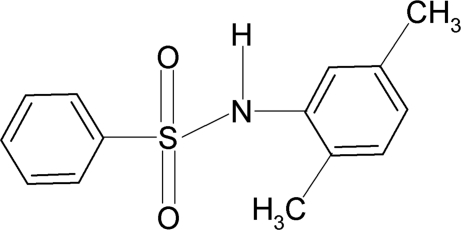


## Experimental

### 

#### Crystal data

C_14_H_15_NO_2_S
*M* *_r_* = 261.33
Monoclinic, 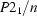

*a* = 10.523 (1) Å
*b* = 8.5631 (7) Å
*c* = 15.135 (2) Å
β = 101.86 (1)°
*V* = 1334.7 (2) Å^3^
*Z* = 4
Mo *K*α radiation
μ = 0.24 mm^−1^
*T* = 299 K
0.44 × 0.40 × 0.34 mm

#### Data collection

Oxford Diffraction Xcalibur diffractometer with Sapphire CCD detector
Absorption correction: multi-scan (*CrysAlis RED*; Oxford Diffraction, 2009 ▶) *T*_min_ = 0.903, *T*_max_ = 0.924
5236 measured reflections
2727 independent reflections
2221 reflections with *I* > 2σ(*I*)
*R*_int_ = 0.010

#### Refinement

*R*[*F* ^2^ > 2σ(*F* ^2^)] = 0.037
*wR*(*F* ^2^) = 0.108
*S* = 1.10
2727 reflections
169 parameters
H atoms treated by a mixture of independent and constrained refinement
Δρ_max_ = 0.27 e Å^−3^
Δρ_min_ = −0.26 e Å^−3^

### 

Data collection: *CrysAlis CCD* (Oxford Diffraction, 2009 ▶); cell refinement: *CrysAlis RED* (Oxford Diffraction, 2009 ▶); data reduction: *CrysAlis RED*; program(s) used to solve structure: *SHELXS97* (Sheldrick, 2008 ▶); program(s) used to refine structure: *SHELXL97* (Sheldrick, 2008 ▶); molecular graphics: *PLATON* (Spek, 2009 ▶); software used to prepare material for publication: *SHELXL97*.

## Supplementary Material

Crystal structure: contains datablocks I, global. DOI: 10.1107/S1600536809041841/pk2200sup1.cif

Structure factors: contains datablocks I. DOI: 10.1107/S1600536809041841/pk2200Isup2.hkl

Additional supplementary materials:  crystallographic information; 3D view; checkCIF report

## Figures and Tables

**Table 1:** Hydrogen-bond geometry (Å, °)

*D*—H⋯*A*	*D*—H	H⋯*A*	*D*⋯*A*	*D*—H⋯*A*
N1—H1*N*⋯O1^i^	0.828 (19)	2.16 (2)	2.9634 (19)	163.3 (19)

Symmetry code: (i) 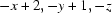.

**Fig. 1.**
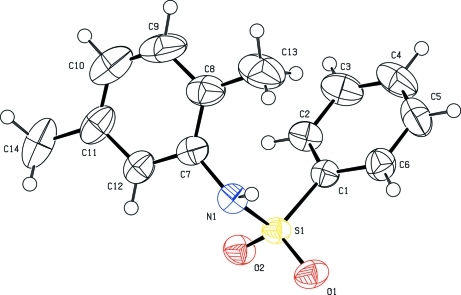
Molecular structure of (I), showing the atom labelling scheme and displacement ellipsoids are drawn at the 50% probability level.

**Fig. 2.**
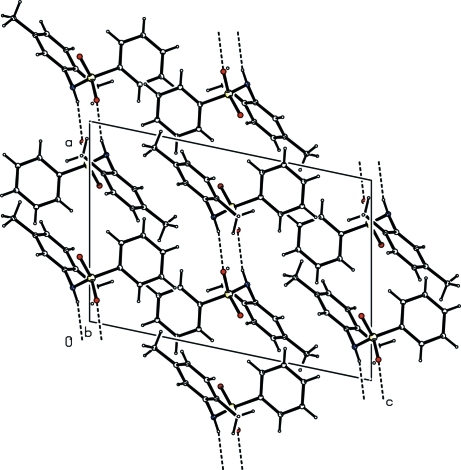
Molecular packing of (I) with hydrogen bonding shown as dashed lines, viewed down the b axis.

## supplementary crystallographic information

### Comment

In the present work, as part of a study of substituent effects on the structures of *N*-(aryl)-arylsulfonamides, the structure of *N*-(2,5-dimethylphenyl)benzenesulfonamide (I) has been determined (Gowda *et al.*, 2008*a*, 2008*b*, 2009). The conformation of the N—C bond in the C—SO_2_—NH—C segment has *gauche* torsions with the S=O bonds (Fig. 1). The molecule is bent at the S atom with the C—SO_2_—NH—C torsion angle of 62.7 (2)°. The two benzene rings in (I) are tilted relative to each other by 40.4 (1)°, compared to the values of 61.5 (1)° in *N*-(2-methylphenyl)benzenesulfonamide (II) (Gowda *et al.*, 2008*b*), 64.8 (1)° in *N*-(2,3-dimethylphenyl)- benzenesulfonamide (III) (Gowda *et al.*, 2009) and 44.9 (1)° in *N*-(2,6-dimethylphenyl)benzenesulfonamide (IV) (Gowda *et al.*, 2008*a*).  The other bond parameters are similar to those observed in (II), (III), (IV) and other aryl sulfonamides (Perlovich *et al.*, 2006; Gelbrich *et al.*, 2007). The crystal packing of (I) showing dimers linked *via* N—H···O(S) hydrogen bonds (Table 1) is given in Fig.2.

### Experimental

A solution of benzene (10 ml) in chloroform (40 ml) was treated dropwise with chlorosulfonic acid (25 ml) at 0 ° C. After the initial evolution of hydrogen chloride subsided, the reaction mixture was brought to room temperature and poured into crushed ice in a beaker. The chloroform layer was separated, washed with cold water and allowed to evaporate slowly. The residual benzenesulfonylchloride was treated with 2,5-dimethylaniline in the stoichiometric ratio and boiled for ten minutes. The reaction mixture was then cooled to room temperature and added to ice cold water (100 ml). The resultant solid *N*-(2,5-dimethylphenyl)benzenesulfonamide was filtered under suction and washed thoroughly with cold water. It was then recrystallized to constant melting point from dilute ethanol. The purity of the compound was checked and characterized by recording its infrared and NMR spectra (Gowda *et al.*, 2005). The single crystals used in X-ray diffraction studies were grown in ethanolic solution by a slow evaporation at room temperature.

### Refinement

The H atom of the NH group was located in a difference map and its position refined with N—H = 0.83 (2) %A. The other H atoms were positioned with idealized geometry using a riding model with C—H = 0.93–0.96 Å. All H atoms were refined with isotropic displacement parameters (set to 1.2 times of the *U*_eq_ of the parent atom).

### Crystal data

**Table:**

C_14_H_15_NO_2_S	*F*(000) = 552
*M**_r_* = 261.33	*D*_x_ = 1.301 Mg m^−^^3^
Monoclinic, *P*2_1_/*n*	Mo *K*α radiation, λ = 0.71073 Å
Hall symbol: -P 2yn	Cell parameters from 2374 reflections
*a* = 10.523 (1) Å	θ = 2.6–27.8°
*b* = 8.5631 (7) Å	µ = 0.24 mm^−^^1^
*c* = 15.135 (2) Å	*T* = 299 K
β = 101.86 (1)°	Prism, colourless
*V* = 1334.7 (2) Å^3^	0.44 × 0.40 × 0.34 mm
*Z* = 4	

### Data collection

**Table:**

Oxford Diffraction Xcalibur (TM) Single Crystal X-ray Diffractometer with Sapphire CCD Detector.	2727 independent reflections
Radiation source: fine-focus sealed tube	2221 reflections with *I* > 2σ(*I*)
graphite	*R*_int_ = 0.010
Detector resolution: 0 pixels mm^-1^	θ_max_ = 26.4°, θ_min_ = 2.6°
Rotation method data acquisition using ω and phi scans.	*h* = −10→13
Absorption correction: multi-scan (*CrysAlis RED*; Oxford Diffraction, 2009)	*k* = −10→7
*T*_min_ = 0.903, *T*_max_ = 0.924	*l* = −17→18
5236 measured reflections	

### Refinement

**Table:**

Refinement on *F*^2^	Secondary atom site location: difference Fourier map
Least-squares matrix: full	Hydrogen site location: inferred from neighbouring sites
*R*[*F*^2^ > 2σ(*F*^2^)] = 0.037	H atoms treated by a mixture of independent and constrained refinement
*wR*(*F*^2^) = 0.108	*w* = 1/[σ^2^(*F*_o_^2^) + (0.0568*P*)^2^ + 0.2974*P*] where *P* = (*F*_o_^2^ + 2*F*_c_^2^)/3
*S* = 1.10	(Δ/σ)_max_ = 0.001
2727 reflections	Δρ_max_ = 0.27 e Å^−^^3^
169 parameters	Δρ_min_ = −0.25 e Å^−^^3^
0 restraints	Extinction correction: *SHELXL*, Fc^*^=kFc[1+0.001xFc^2^λ^3^/sin(2θ)]^-1/4^
Primary atom site location: structure-invariant direct methods	Extinction coefficient: 0.0149 (18)

### Special details

**Table:**

Experimental. Empirical absorption correction using spherical harmonics, implemented in SCALE3 ABSPACK scaling algorithm
Geometry. All e.s.d.'s (except the e.s.d. in the dihedral angle between two l.s. planes) are estimated using the full covariance matrix. The cell e.s.d.'s are taken into account individually in the estimation of e.s.d.'s in distances, angles and torsion angles; correlations between e.s.d.'s in cell parameters are only used when they are defined by crystal symmetry. An approximate (isotropic) treatment of cell e.s.d.'s is used for estimating e.s.d.'s involving l.s. planes.
Refinement. Refinement of *F*^2^ against ALL reflections. The weighted *R*-factor *wR* and goodness of fit *S* are based on *F*^2^, conventional *R*-factors *R* are based on *F*, with *F* set to zero for negative *F*^2^. The threshold expression of *F*^2^ > σ(*F*^2^) is used only for calculating *R*-factors(gt) *etc*. and is not relevant to the choice of reflections for refinement. *R*-factors based on *F*^2^ are statistically about twice as large as those based on *F*, and *R*- factors based on ALL data will be even larger.

### Fractional atomic coordinates and isotropic or equivalent isotropic displacement parameters (Å^2^)

**Table:**

	*x*	*y*	*z*	*U*_iso_*/*U*_eq_	
S1	0.78193 (4)	0.44572 (5)	−0.00630 (3)	0.03753 (16)	
O1	0.88777 (11)	0.35489 (14)	−0.02510 (9)	0.0504 (3)	
O2	0.68808 (12)	0.37259 (15)	0.03523 (9)	0.0476 (3)	
N1	0.84891 (13)	0.58790 (18)	0.05948 (10)	0.0417 (4)	
H1N	0.9148 (19)	0.619 (2)	0.0428 (13)	0.050*	
C1	0.70021 (15)	0.53338 (19)	−0.10743 (11)	0.0381 (4)	
C2	0.57570 (16)	0.5897 (2)	−0.11257 (13)	0.0474 (4)	
H2	0.5335	0.5753	−0.0649	0.057*	
C3	0.5152 (2)	0.6672 (3)	−0.18942 (16)	0.0647 (6)	
H3	0.4315	0.7057	−0.1937	0.078*	
C4	0.5778 (2)	0.6880 (3)	−0.25983 (16)	0.0722 (7)	
H4	0.5361	0.7404	−0.3115	0.087*	
C5	0.7015 (2)	0.6322 (3)	−0.25444 (14)	0.0672 (6)	
H5	0.7429	0.6467	−0.3025	0.081*	
C6	0.76449 (19)	0.5545 (2)	−0.17803 (13)	0.0513 (5)	
H6	0.8484	0.5170	−0.1739	0.062*	
C7	0.76772 (16)	0.7043 (2)	0.08868 (12)	0.0437 (4)	
C8	0.7529 (2)	0.8517 (2)	0.04985 (15)	0.0607 (5)	
C9	0.6695 (3)	0.9543 (3)	0.0839 (2)	0.0831 (8)	
H9	0.6550	1.0538	0.0593	0.100*	
C10	0.6091 (3)	0.9108 (3)	0.1524 (2)	0.0825 (8)	
H10	0.5540	0.9815	0.1722	0.099*	
C11	0.62731 (19)	0.7668 (3)	0.19244 (15)	0.0622 (6)	
C12	0.70773 (16)	0.6642 (2)	0.15926 (13)	0.0486 (4)	
H12	0.7221	0.5655	0.1849	0.058*	
C13	0.8207 (3)	0.9011 (3)	−0.0238 (2)	0.0899 (8)	
H13A	0.9091	0.8655	−0.0099	0.108*	
H13B	0.8193	1.0129	−0.0285	0.108*	
H13C	0.7771	0.8565	−0.0801	0.108*	
C14	0.5648 (2)	0.7236 (4)	0.27002 (18)	0.0873 (9)	
H14A	0.5622	0.8136	0.3074	0.105*	
H14B	0.6143	0.6425	0.3050	0.105*	
H14C	0.4780	0.6873	0.2471	0.105*	

### Atomic displacement parameters (Å^2^)

**Table:**

	*U*^11^	*U*^22^	*U*^33^	*U*^12^	*U*^13^	*U*^23^
S1	0.0331 (2)	0.0359 (2)	0.0443 (3)	0.00200 (16)	0.00952 (16)	0.00318 (17)
O1	0.0408 (7)	0.0461 (7)	0.0665 (8)	0.0092 (5)	0.0160 (6)	0.0016 (6)
O2	0.0451 (7)	0.0453 (7)	0.0557 (7)	−0.0032 (5)	0.0178 (6)	0.0076 (6)
N1	0.0323 (7)	0.0464 (8)	0.0455 (8)	−0.0026 (6)	0.0059 (6)	0.0000 (6)
C1	0.0367 (8)	0.0365 (9)	0.0401 (9)	−0.0039 (7)	0.0057 (6)	−0.0013 (7)
C2	0.0361 (9)	0.0486 (10)	0.0563 (11)	−0.0009 (8)	0.0065 (7)	0.0057 (8)
C3	0.0464 (11)	0.0637 (13)	0.0759 (15)	−0.0004 (10)	−0.0062 (10)	0.0161 (11)
C4	0.0747 (15)	0.0708 (15)	0.0588 (13)	−0.0115 (12)	−0.0145 (11)	0.0211 (11)
C5	0.0848 (16)	0.0733 (15)	0.0435 (11)	−0.0153 (13)	0.0134 (10)	0.0075 (10)
C6	0.0522 (10)	0.0533 (11)	0.0513 (11)	−0.0013 (9)	0.0174 (8)	0.0003 (9)
C7	0.0362 (8)	0.0413 (9)	0.0479 (10)	−0.0017 (7)	−0.0048 (7)	−0.0071 (8)
C8	0.0646 (13)	0.0414 (10)	0.0685 (14)	−0.0071 (9)	−0.0041 (10)	−0.0037 (9)
C9	0.0940 (19)	0.0369 (11)	0.105 (2)	0.0072 (12)	−0.0096 (16)	−0.0086 (12)
C10	0.0697 (15)	0.0679 (16)	0.106 (2)	0.0097 (13)	0.0097 (14)	−0.0323 (15)
C11	0.0484 (11)	0.0638 (14)	0.0715 (14)	−0.0028 (10)	0.0057 (9)	−0.0311 (11)
C12	0.0402 (9)	0.0514 (11)	0.0507 (10)	−0.0020 (8)	0.0016 (7)	−0.0129 (8)
C13	0.113 (2)	0.0534 (14)	0.097 (2)	−0.0199 (15)	0.0091 (16)	0.0187 (13)
C14	0.0714 (15)	0.109 (2)	0.0881 (18)	−0.0067 (15)	0.0315 (13)	−0.0484 (16)

### Geometric parameters (Å, °)

**Table:**

S1—O2	1.4205 (12)	C7—C8	1.387 (3)
S1—O1	1.4340 (12)	C7—C12	1.391 (3)
S1—N1	1.6377 (15)	C8—C9	1.412 (3)
S1—C1	1.7620 (17)	C8—C13	1.503 (4)
N1—C7	1.440 (2)	C9—C10	1.374 (4)
N1—H1N	0.828 (19)	C9—H9	0.9300
C1—C2	1.383 (2)	C10—C11	1.370 (4)
C1—C6	1.389 (2)	C10—H10	0.9300
C2—C3	1.376 (3)	C11—C12	1.384 (3)
C2—H2	0.9300	C11—C14	1.505 (3)
C3—C4	1.375 (3)	C12—H12	0.9300
C3—H3	0.9300	C13—H13A	0.9600
C4—C5	1.373 (3)	C13—H13B	0.9600
C4—H4	0.9300	C13—H13C	0.9600
C5—C6	1.380 (3)	C14—H14A	0.9600
C5—H5	0.9300	C14—H14B	0.9600
C6—H6	0.9300	C14—H14C	0.9600
			
O2—S1—O1	119.19 (8)	C12—C7—N1	117.14 (16)
O2—S1—N1	108.07 (8)	C7—C8—C9	115.8 (2)
O1—S1—N1	105.61 (8)	C7—C8—C13	122.8 (2)
O2—S1—C1	108.22 (8)	C9—C8—C13	121.4 (2)
O1—S1—C1	108.61 (8)	C10—C9—C8	121.7 (2)
N1—S1—C1	106.47 (8)	C10—C9—H9	119.2
C7—N1—S1	119.53 (11)	C8—C9—H9	119.2
C7—N1—H1N	117.4 (15)	C11—C10—C9	122.3 (2)
S1—N1—H1N	109.5 (14)	C11—C10—H10	118.9
C2—C1—C6	121.13 (16)	C9—C10—H10	118.9
C2—C1—S1	119.01 (13)	C10—C11—C12	116.8 (2)
C6—C1—S1	119.72 (13)	C10—C11—C14	121.5 (2)
C3—C2—C1	118.92 (18)	C12—C11—C14	121.7 (2)
C3—C2—H2	120.5	C11—C12—C7	122.0 (2)
C1—C2—H2	120.5	C11—C12—H12	119.0
C4—C3—C2	120.4 (2)	C7—C12—H12	119.0
C4—C3—H3	119.8	C8—C13—H13A	109.5
C2—C3—H3	119.8	C8—C13—H13B	109.5
C5—C4—C3	120.5 (2)	H13A—C13—H13B	109.5
C5—C4—H4	119.7	C8—C13—H13C	109.5
C3—C4—H4	119.7	H13A—C13—H13C	109.5
C4—C5—C6	120.3 (2)	H13B—C13—H13C	109.5
C4—C5—H5	119.9	C11—C14—H14A	109.5
C6—C5—H5	119.9	C11—C14—H14B	109.5
C5—C6—C1	118.76 (19)	H14A—C14—H14B	109.5
C5—C6—H6	120.6	C11—C14—H14C	109.5
C1—C6—H6	120.6	H14A—C14—H14C	109.5
C8—C7—C12	121.37 (18)	H14B—C14—H14C	109.5
C8—C7—N1	121.47 (18)		
			
O2—S1—N1—C7	−53.36 (15)	S1—C1—C6—C5	−175.98 (16)
O1—S1—N1—C7	178.05 (13)	S1—N1—C7—C8	−102.82 (18)
C1—S1—N1—C7	62.70 (15)	S1—N1—C7—C12	78.98 (18)
O2—S1—C1—C2	31.30 (16)	C12—C7—C8—C9	−2.6 (3)
O1—S1—C1—C2	162.03 (14)	N1—C7—C8—C9	179.28 (17)
N1—S1—C1—C2	−84.66 (15)	C12—C7—C8—C13	177.4 (2)
O2—S1—C1—C6	−153.10 (14)	N1—C7—C8—C13	−0.8 (3)
O1—S1—C1—C6	−22.37 (16)	C7—C8—C9—C10	1.2 (3)
N1—S1—C1—C6	90.94 (15)	C13—C8—C9—C10	−178.8 (2)
C6—C1—C2—C3	0.2 (3)	C8—C9—C10—C11	0.8 (4)
S1—C1—C2—C3	175.78 (15)	C9—C10—C11—C12	−1.5 (3)
C1—C2—C3—C4	0.1 (3)	C9—C10—C11—C14	177.4 (2)
C2—C3—C4—C5	−0.1 (4)	C10—C11—C12—C7	0.0 (3)
C3—C4—C5—C6	−0.1 (4)	C14—C11—C12—C7	−178.80 (18)
C4—C5—C6—C1	0.4 (3)	C8—C7—C12—C11	2.1 (3)
C2—C1—C6—C5	−0.5 (3)	N1—C7—C12—C11	−179.74 (15)

### Hydrogen-bond geometry (Å, °)

**Table:**

*D*—H···*A*	*D*—H	H···*A*	*D*···*A*	*D*—H···*A*
N1—H1N···O1^i^	0.828 (19)	2.16 (2)	2.9634 (19)	163.3 (19)

Symmetry codes: (i) −*x*+2, −*y*+1, −*z*.

## References

[bb1] Gelbrich, T., Hursthouse, M. B. & Threlfall, T. L. (2007). *Acta Cryst.* B**63**, 621–632. 10.1107/S010876810701395X 17641433

[bb2] Gowda, B. T., Foro, S., Babitha, K. S. & Fuess, H. (2008*a*). *Acta Cryst.* E**64**, o1691. 10.1107/S1600536808024653 PMC2960643 21201680

[bb3] Gowda, B. T., Foro, S., Babitha, K. S. & Fuess, H. (2008*b*). *Acta Cryst.* E**64**, o1692. 10.1107/S1600536808024562 PMC2960504 21201681

[bb4] Gowda, B. T., Foro, S., Babitha, K. S. & Fuess, H. (2009). *Acta Cryst.* E**65**, o366. 10.1107/S1600536809002098 PMC2968343 21581964

[bb5] Gowda, B. T., Shetty, M. & Jayalakshmi, K. L. (2005). *Z. Naturforsch. Teil A*, **60**, 106–112.

[bb6] Oxford Diffraction (2009). *CrysAlis CCD* and *CrysAlis RED* Oxford Diffraction Ltd, Abingdon, England.

[bb7] Perlovich, G. L., Tkachev, V. V., Schaper, K.-J. & Raevsky, O. A. (2006). *Acta Cryst.* E**62**, o780–o782.

[bb8] Sheldrick, G. M. (2008). *Acta Cryst.* A**64**, 112–122. 10.1107/S0108767307043930 18156677

[bb9] Spek, A. L. (2009). *Acta Cryst.* D**65**, 148–155. 10.1107/S090744490804362X PMC2631630 19171970

